# DDOST is associated with tumor immunosuppressive microenvironment in cervical cancer

**DOI:** 10.1007/s12672-024-00927-z

**Published:** 2024-03-09

**Authors:** Jie Mei, Liuliu Pan, Min Huang, Dandan Bao, Hui Gao, Danhan Wang

**Affiliations:** 1 Department of Obstetrics and Gynecology, The Second Affiliated Hospital of Wenzhou Medical University, Wenzhou, 325027 China; 2 Key Laboratory of Fertility Preservation and Maintenance of Ministry of Education, Ningxia Medical University, Yinchuan, 750004 China

**Keywords:** Cervical cancer, DDOST, TME, Biomarker, Treatment

## Abstract

Evidence has revealed that DDOST plays an important role in cancer development and progression. However, there are no reports on functions of DDOST in cervical tumorigenesis. Hence, we investigated the relationship of DDOST with prognosis, mutation, promoter methylation, immune cell infiltration, and drug sensitivity using bioinformatics techniques. Our results demonstrated that DDOST was significantly upregulated in a variety of tumor types and correlated with poor prognosis, including cervical cancer. Cox regression analysis dissected that high DDOST expression was associated with poor survival in cervical cancer patients. Immune infiltration analysis defined that DDOST was negatively correlated with CD8 T cells and NK cells. Strikingly, the sensitivity to multiple drugs was negatively correlated with the expression of DDOST. Therefore, our findings uncovered that DDOST could play an essential role in the tumor microenvironment and tumor immune regulation in cervical cancer, which indicated that DDOST could be a useful biomarker for prognosis and a potential therapeutic target for cancer treatment.

**Supplementary Information:**

The online version contains supplementary material available at 10.1007/s12672-024-00927-z.

## Introduction

Cervical cancer is one of the common gynecological cancers [1]. The number of patients who die each year due to cervical cancer exceeds 260,000, making cervical cancer the second most lethal malignancy in women in the world [2]. Human papillomavirus (HPV) infection has been found to be associated with cervical cancer developed [3]. The treatments include surgery, chemotherapy, and immunotherapy. Chemotherapy agents for cervical cancer often include cisplatin, paclitaxel, and topotecan [4]. Cervical oncogenesis is correlated with dysregulation of genetics and epigenetics, including noncoding RNAs [5].

Despite precancerous screening and emerging treatment options, cervical cancer remains one of the leading causes of death among women in developing countries [6–8]. When cervical cancer patients present with metastasis and recurrence, their prognosis is worse [9]. Therefore, based on its molecular mechanisms that remain to be elucidated, the search for new prognostic markers as well as the development of new therapeutic approaches is of great significance for cervical cancer [10–12].

DDOST (dolichyl-diphosphooligosaccharide–protein glycosyltransferase non-catalytic subunit), encodes a component of the oligosaccharide transferase (OST) complex and is related to the N-glycosylation of proteins [13]. DDOST was reported to be not associated with diabetic nephropathy in type 1 diabetes [14]. DDOST expression was regulated by dietary advanced glycated end-products and statins and angiotensin receptor inhibitors in peripheral mononuclear cells from patients with type 1 diabetes [15]. In congenital disorders of glycosylation, whole-exome sequencing discovered the mutations of DDOST [16]. Furthermore, DDOST was identified to interact with PPP1CC2 in transgenic mouse embryonic stem cells [17]. Several studies revealed that DDOST might be an oncogene in several tumor types. In esophageal squamous cell carcinoma (ESCC) patients, DDOST displayed missense variants, indicating that these variants might be diagnostic biomarkers for ESCC diagnosis [18]. Using integrated analysis of gene expression and DNA methylation profiles, one study found that DDOST gene was one of core genes in bladder cancer development [19]. However, the role of DDOST in cervical cancer was still unclear. Thus, we aim to explore the function of DDOST in cervical tumorigenesis.

Our study utilized multiple publicly available data to perform a systematic analysis of the pan-cancer role of DDOST in tumorigenesis. The underlying molecular mechanisms of DDOST in cervical cancer as well as pan-cancer differential expression, prognosis, genetic alterations, immune infiltration and sensitivity to targeted agents were explored. Quantitative real-time PCR assay and immunohistochemistry (IHC) were performed to validate the role of DDOST in cervical cancer. This study provides a theoretical basis for gaining insight into the role of DDOST in tumors.

## Materials and methods

### Differential expression analysis and data processing

The expression information of DDOST in 31 types of normal tissues was obtained from the GTEx portal, and the expression of DDOST was compared between 33 types of cancer tissues and 31 types of normal tissues (no normal tissues available for MESO and UVM, MESO: mesothelioma, UVM: uveal melanoma) by combining the data from TCGA with the data from GTEx. Both TCGA and GTEX data were downloaded from UCSC Xena database (https://xenabrowser.net/datapages/), tumor tissues were obtained from TCGA database, and normal tissues were derived from TCGA database and GTEx database.

### Survival analysis

We obtained data from TCGA database to reveal the correlation of DDOST expression with patient prognosis, mainly in relation to overall survival (OS), disease-free interval (DFI), progression-free interval (PFI), and disease-specific survival (DSS). We analyzed the survival of all 33 cancer types, and the results were shown by forest plots and Kaplan–Meier curves from the univariate Cox regression analysis. The survminer R packages and survival were used for analysis and visualization.

### Mutation analysis of the DDOST gene

The cbioportal database was selected for DDOST gene mutation analysis. Through this website, we obtained information on DDOST gene mutation frequency, mutation type, copy number alterations, and promoter methylation in pan-cancer tissues, including cervical cancer, from TCGA database, using human methylation 450 K array methylation data.

### Enrichment analysis of DDOST

We leveraged GSEA to analyze the underlying molecular mechanism of DDOST in 33 cancer types. Analysis and visualization were performed using the R package clusterProfiler. We downloaded 50 HALLMARK pathways from the MsigDB database (http://www.gsea-msigdb.org/gsea/msigdb/index.jsp) and used GSVA package to score HALLMARK pathways and calculate the correlation of DDOST expression with pathways in pan-cancer, including cervical cancer.

### Tumor microenvironment analysis

Tumor microenvironment analysis for each tumor demonstrates the fraction of pathways differentially represented in the high and low DDOST gene expression groups, and a heatmap was used to demonstrate the correlation of genes with pathway scores [20]. The correlation between DDOST expression and the TME-related signatures in pan-cancer was explored. From the ImmuCellAI database (bioinfo.life.hust.edu.cn/web/ImmuCellAI/), we downloaded pan-cancer immune infiltration data. The correlation of DDOST expression with immune cell infiltration was calculated and displayed.

### DDOST related drug sensitivity analysis

Using GDSC (https://www.cancerrxgene.org/) database, we downloaded the half maximal inhibitory concentration (IC50) and gene expression data of tumor cells, analyzed the relationship between DDOST and drug (IC50), including ABT737, WEHI-539, Sabutoclax, PF-4708671, KU-55933, and Trametinib, and plotted the correlation between the expression of DDOST in each drug and IC50, respectively.

### Cell culture

CaSki (HPV16), SiHa (HPV16), HeLa (HPV18), C33A (HPV negative) and Ect1/E6E7 cells were purchased from the American Type Culture Collection. HeLa, SiHa and Ect1/E6E7 cells were cultured in DMEM medium. C33A and CaSki cells were cultures in RPMI 1640 medium. Cell medium were supplemented with 10% FBS and 1% penicillin/streptomycin. Cells were maintained in 37 °C incubator with 5% CO_2._

### Quantitative real-time PCR assay

Total RNA (1 g) was extracted from cells using RNA-easy Isolation Reagent (R701-01, Vazyme) following the manufacturer’s instructions. Reverse transcription was performed using TransScript All-in-One First-Strand cDNA Synthesis SuperMix for qPCR (P20607, TransGen Biotech, China), and the reaction was blended with PerfectStart Green qPCR SuperMix (P20604,TransGen Biotech, China) and run on a BIO-RAD CFX96 Real time PCR system as follows: 94 °C 30 s, 94 °C 5 s and 60 °C 30 s for 40 cycles. The sequences of primers were: DDOST: forward 5′-GCT CAC ATT CAA GAC CGC TG-3′; reverse 5′-CGT GAT CCA GCA GCT CTC AA-3′. Beta actin: forward 5′-ACA GAG CCT CGC CTT TGC CGA T-3′; reverse 5′-GGC CTC GTC GCC CAC ATA GGA-3′. Quantitative real-time PCR was conducted as described previously [21].

### Immunohistochemistry (IHC)

We collected 40 cervical cancer tissues and their adjacent non-tumor tissues. The tissues were formalin-fixed, paraffin-embedded, and sliced into 4 M section. We used xylene to deparaffinize and rehydrated in ethanol. Then, microwave was used to retrieve antigen, and anti-DDOST antibody (1:500, Rabbit polyclonal antibody, Abcam Company, ab204314) was applied for incubation with the slices overnight at 4 °C. The slices were washed by PBS three times, and incubated with the relevant secondary antibody for 45 min. Then, IHC were conducted as described previously [22]. The human tissue study was approved by the ethical committee of the Second Affiliated Hospital of Wenzhou Medical University.

### Statistical analysis

The GraphPad Prism 7.0 software was used for statistical analysis. The difference between two groups was analyzed with the Student’s t test. The difference among multiple groups was analyzed by ANOVA (Analysis of variance). P < 0.05 was considered significant. All data are presented as mean ± standard deviation (SD).

## Results

### Expression levels of DDOST in multiple cancer types and normal tissues

**Fig. 1:**
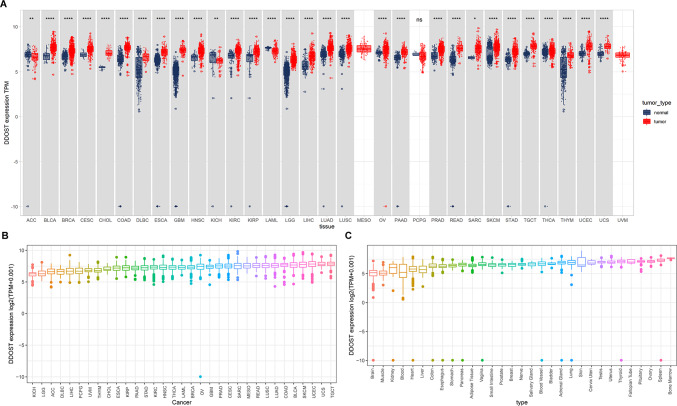
DDOST expression analysis in Pan-cancer. **A**, Pan-cancer differential expression of DDOST between tumor tissues from TCGA and normal tissues from TCGA and GTEx database is illustrated. **B**, DDOST expression in tumor tissues from TCGA database. **C**, DDOST expression in normal tissues from GTEx database. *P < 0.05, **P < 0.01, ***P < 0.001, ****P < 0.0001

We first performed a joint analysis of TCGA and GTEx data and found that DDOST expression was differed between 30 types of cancer samples and normal tissues. Among them, DDOST was highly expressed in 27 cancer types, including CESC, and lowly expressed in 3 cancer types compared with corresponding normal tissues (Fig. 1A). Based on the TCGA database, we analyzed the expression levels of DDOST in tumor tissues of 33 cancer types and ranked them from high to low by mean expression of DDOST (Fig. 1B). All cancer types expressed DDOST, with the highest level of expression in TGCT and the lowest in KICH. Next, we analyzed the DDOST expression levels among normal tissues using the GTEx data. Expression level of DDOST was highest in bone marrow and lowest in brain (Fig. 1C).

**Fig. 2:**
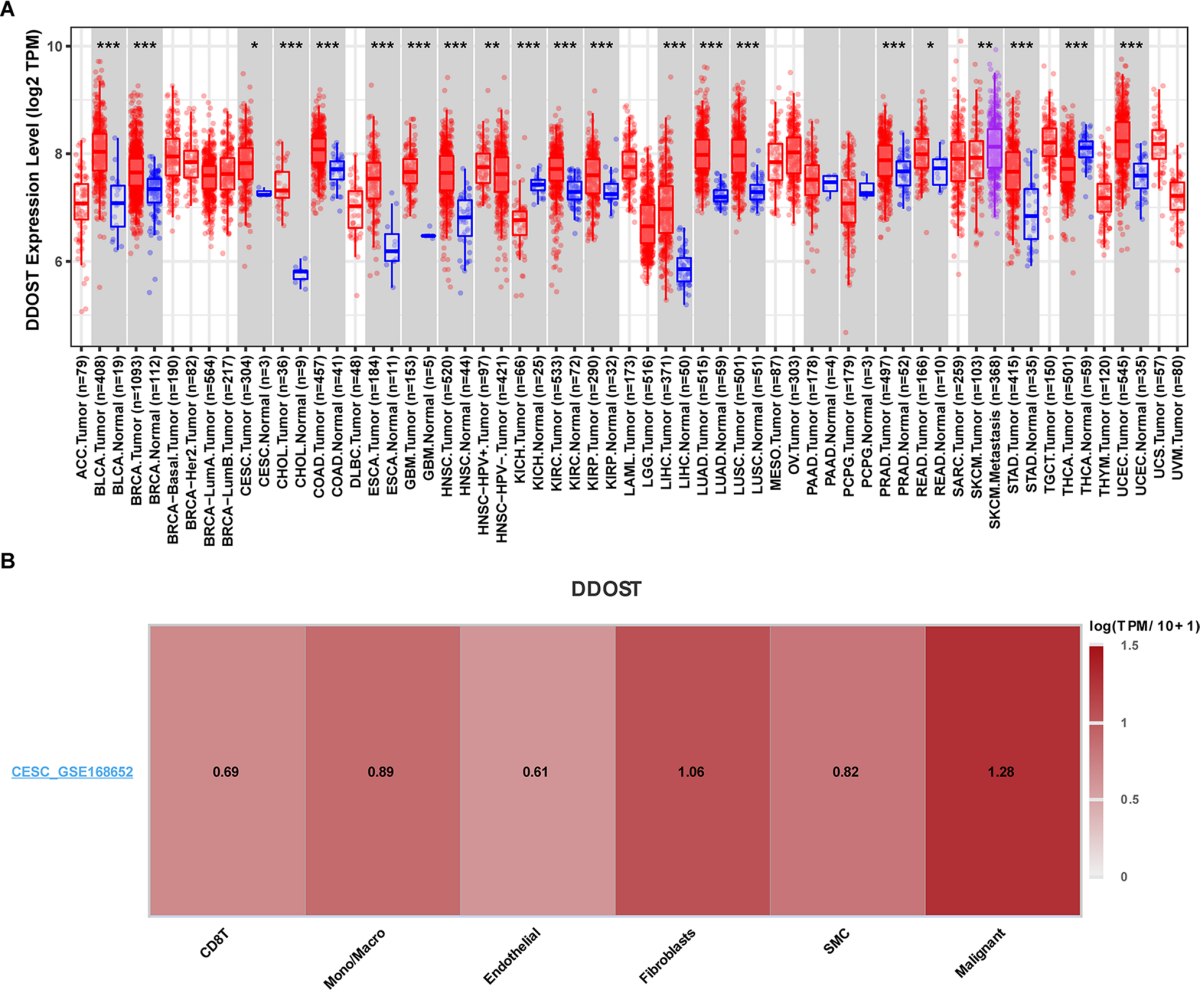
DDOST expression in single cell sequence data. **A**. The differential expression of DDOST between tumor tissues and normal tissues was presented. **B**. DDOST expression in single cell sequence data of cervical cancer using TISCH database. *P < 0.05, **P < 0.01, ***P < 0.001

We also analyzed the DDOST expression based on TCGA data and found that DDOST was overexpressed in most tumor types, including CESC (Fig. 2A). Using single cell expression profiling of cervical cancer, we revealed that DDOST was universally expressed in tumor microenvironment (TME), including CD8 T cells, fibroblasts and SMC. The most highly expression of DDOST was in malignant cells (Fig. 2B).

### Correlation analysis between DDOST expression and gene alterations

**Fig. 3:**
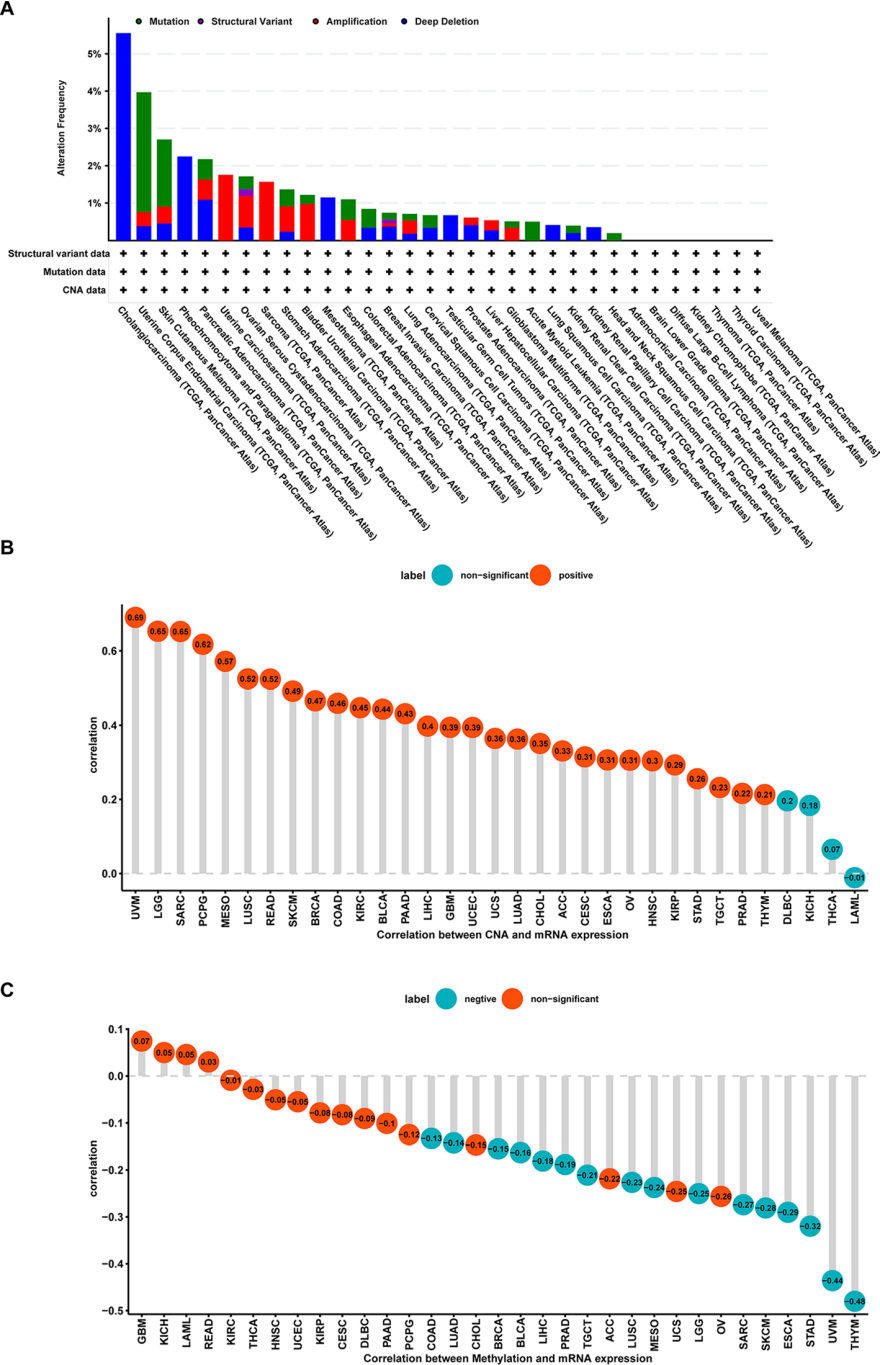
The gene alteration of DDOST in pan-cancer. **A**, CNA and mutation frequency of DDOST in TCGA pan-cancer were accessed from cBioPortal database. **B**, The correlation of DDOST mRNA expression and linear copy-number value in indicated tumor types from TCGA. **C**, The correlation of DDOST mRNA expression and promoter methylation level in indicated tumor types from TCGA

We further evaluated the gene variation landscape of DDOST in different tumor samples, including mutation, amplification, and deletion types, through the cBioportal database. As shown in Fig. 3A, DDOST had the highest alteration frequency (> 5%) in patients with CHOL, which was predominantly “Deep Deletion”. The correlation of DDOST expression with copy number was shown in Fig. 3B. In cervical cancer, DDOST expression was positively correlated with copy number. Correlation between DDOST gene expression and promoter methylation was revealed, and there was no significant correlation between DDOST expression and its methylation in cervical cancer (Fig. 3C), suggesting that hypomethylation of DDOST promoter could lead to high expression of DDOST in cervical cancer.

### Pan-cancer analysis of the prognostic value of DDOST

**Fig. 4:**
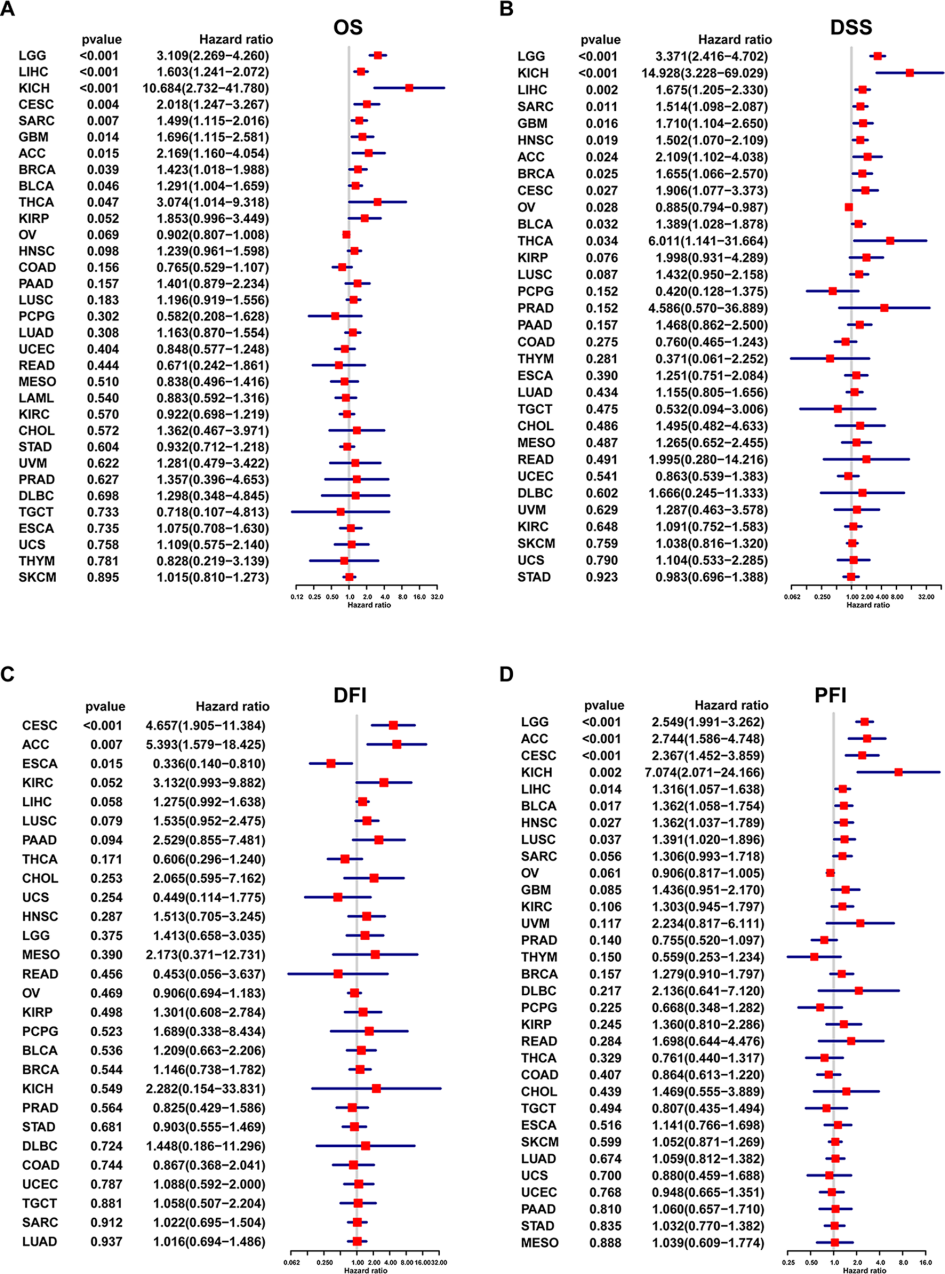
Univariate regression analysis of DDOST in pan-cancer. **A**–**D**, Forest map shows the univariate cox regression analysis results of DDOST in TCGA pan-cancer, including OS (**A**), DSS (**B**), DFI (**C**), and PFI (**D**)

**Fig. 5:**
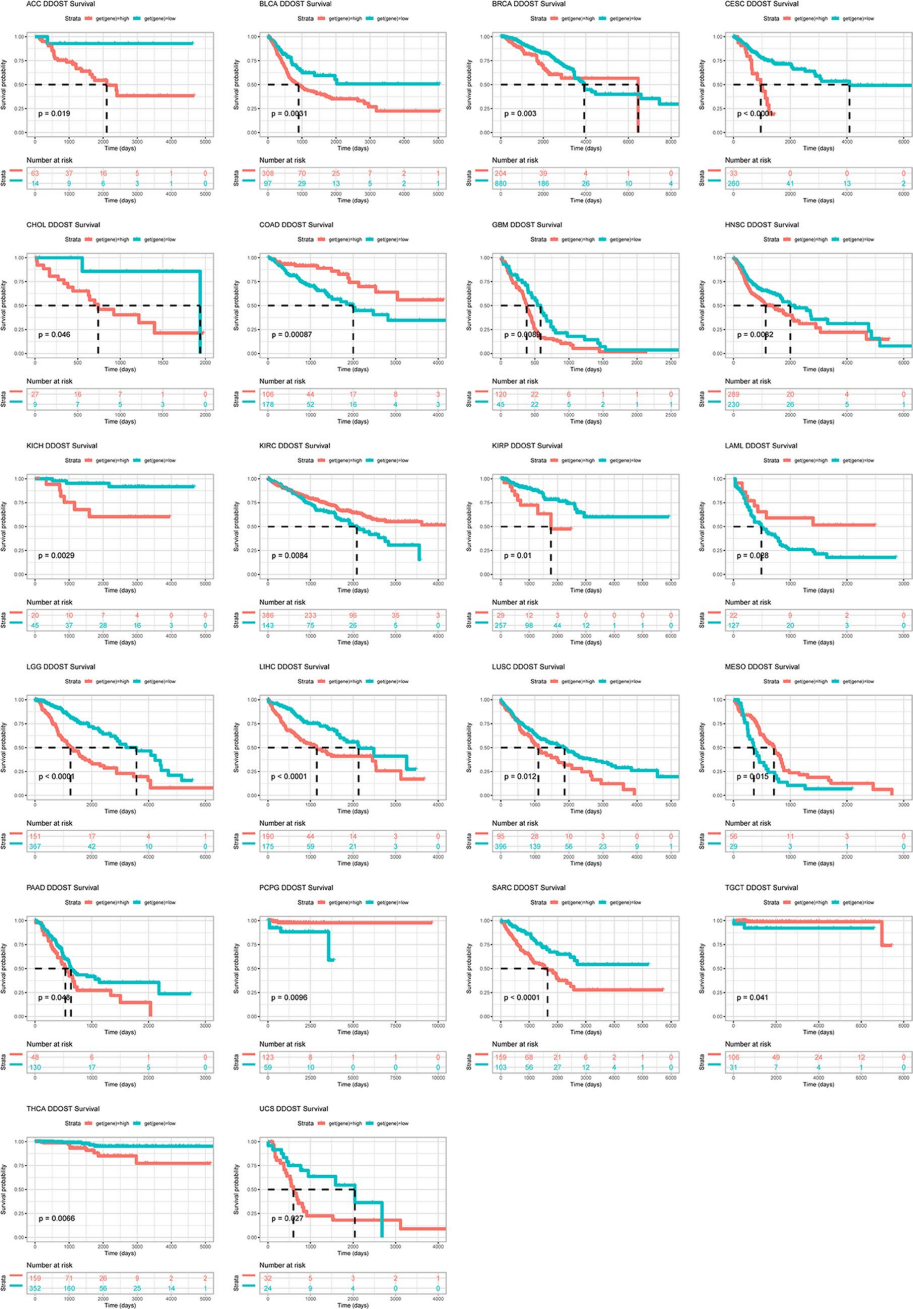
Overall survival analysis of DDOST in pan-cancer. Kaplan–Meier survival analysis results of DDOST in indicated tumor types from TCGA pan-cancer

We evaluated the relationship between DDOST expression and patient prognosis in a pan-cancer, with survival metrics as OS, DSS, DFI, and PFI. Cox regression analysis of 33 tumor types showed that the expression of DDOST was significantly associated with prognosis in multiple cancer types, especially cervical cancer. Cervical cancer patients with high expression of DDOST had worse OS (Fig. 4A), DSS (Fig. 4B), DFI (Fig. 4C), and PFI (Fig. 4D). Furthermore, Kaplan–Meier survival curves showed that in 16 cancer types, DDOST was a high-risk factor for shorter OS, including cervical cancer (Fig. 5).

### Enrichment analysis of DDOST

**Fig. 6:**
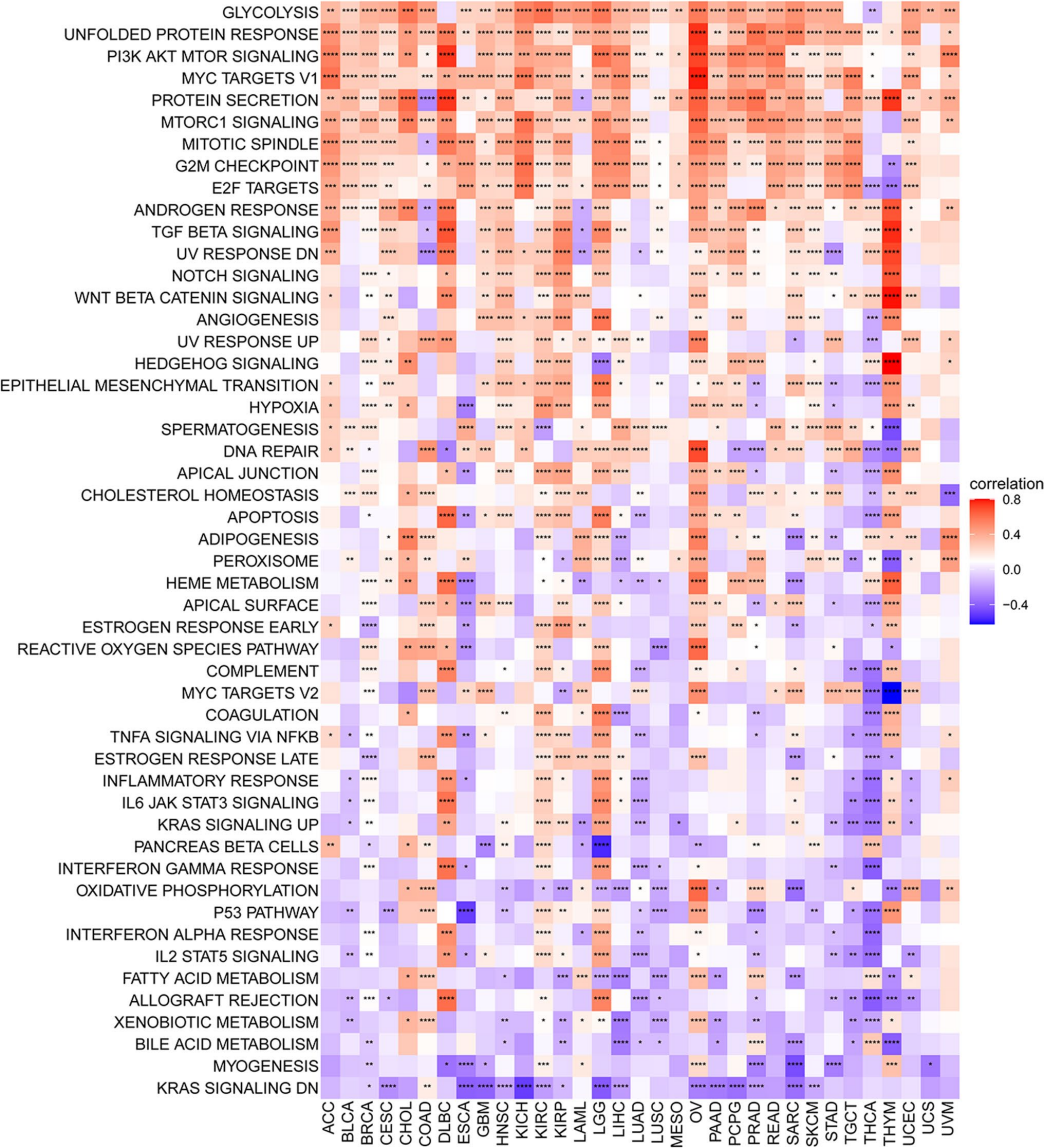
GSVA of DDOST. The GSVA results of DDOST based on 50 HALLMARK pathways in pan-cancer. *P < 0.05, **P < 0.01, ***P < 0.001, ****P < 0.0001

**Fig. 7:**
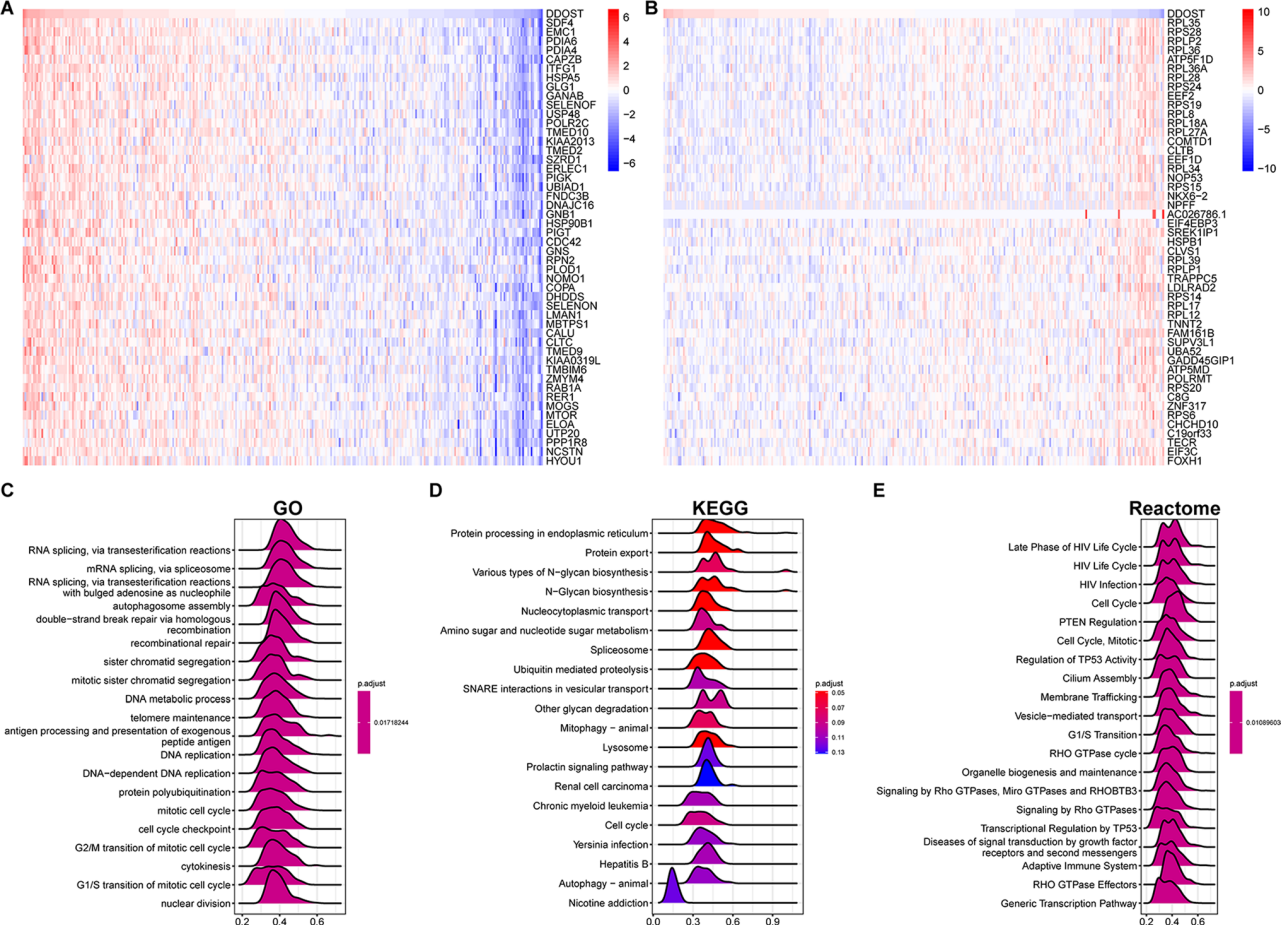
GSEA of DDOST. **A**–**B**, The expression of top 50 genes positively or negatively associated with DDOST expression in cervical cancer. **C**–**E** The top 20 GSEA results of DDOST were showed based on GO (**C**), KEGG (**D**), and Reactome (**E**) pathways in cervical cancer

To explore the biological significance of DDOST in different tumor tissues, we analyzed the correlation between DDOST expression and 50 HALLMARK pathways. We found that DDOST was most strongly associated with Glycolysis, Unfolded protein response, and PI3K/AKT/mTOR signaling (Fig. 6). We next performed correlation analysis of DDOST with all mRNAs in cervical cancer and demonstrated gene expression of Top50 positively (Fig. 7A) and negatively (Fig. 7B) correlated with DDOST, respectively. DDOST was closely associated with RNA splicing via transesterification reactions in GO terms (Fig. 7C), protein processing in endoplasmic reticulum in KEGG terms (Fig. 7D), and late phase of HIV life cycle and most cell cycle-related pathways in Reactome terms (Fig. 7E) in cervical cancer.

### Association of DDOST with TME

**Fig. 8:**
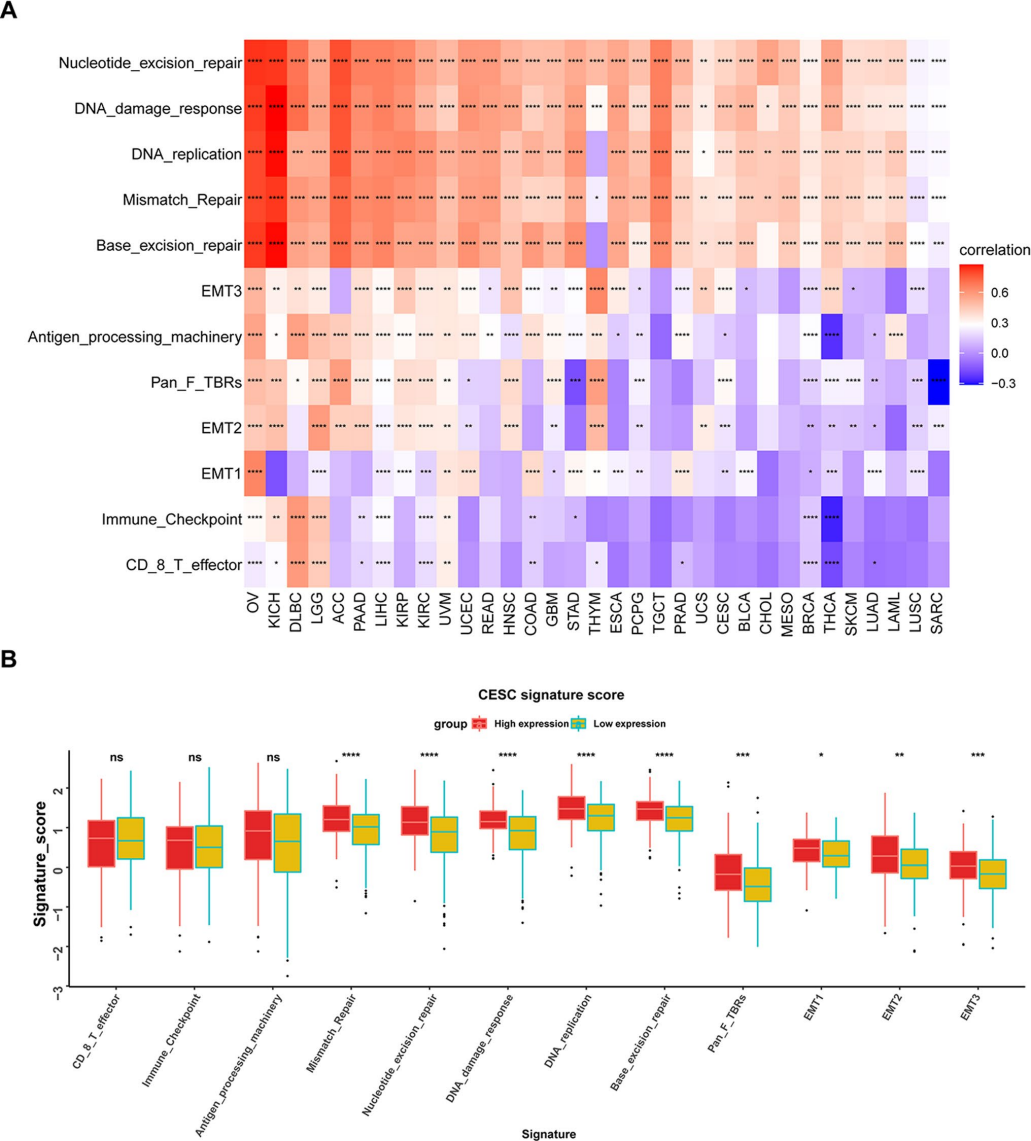
TME analysis of DDOST. **A**. The correlation of DDOST with indicated TME-related pathways in pan-cancer. **B**. The score of indicated TME-related pathways in high- and low-DDOST expression group in cervical cancer. *P < 0.05, **P < 0.01, ***P < 0.001, ****P < 0.0001

Next, we explored the correlation between DDOST expression and the TME-related signatures in pan-cancer. As shown in Fig. 8A, DDOST expression was significantly correlated with various pathway scores in pan-cancer, among which were mainly DNA damage repair and EMT related pathways. In cervical cancer, DNA damage repair and EMT related pathways were enriched in DDOST high-expression group (Fig. 8B).

**Fig. 9:**
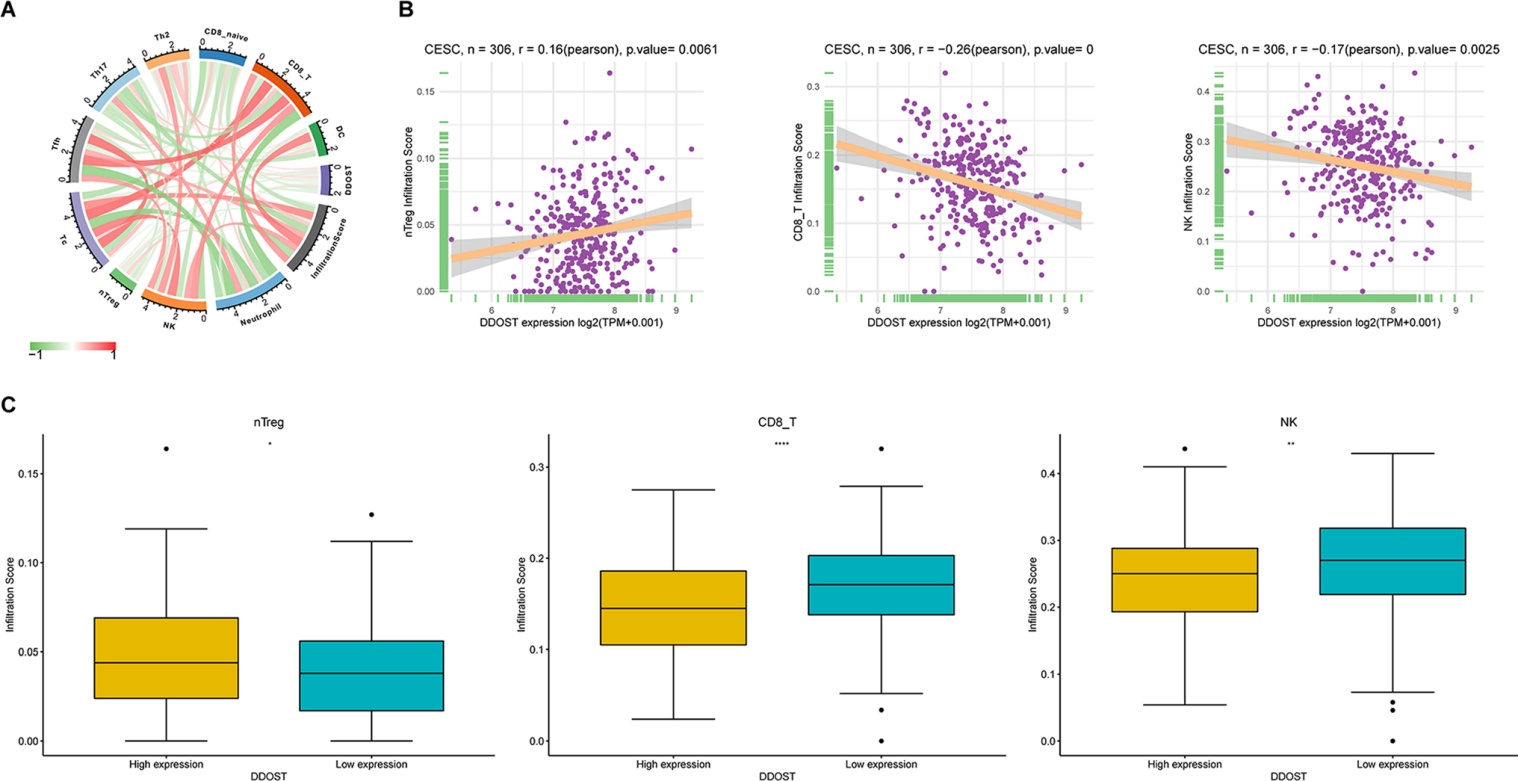
Immune infiltration analysis**.** **A–B**, The relationship between DDOST expression and infiltration levels of indicated immune cells in cervical cancer. **C**. The infiltration levels of indicated immune cells in high- and low-DDOST expression group in cervical cancer. *P < 0.05, **P < 0.01, ****P < 0.0001. P = 0 means p < 0.0001

We also analyzed the correlation of DDOST with immune cell infiltration in the TME of cervical cancer. A total of 10 immune cells were related with DDOST (Fig. 9A). DDOST expression in cervical cancer was positively correlated with cancer-promoting immune cell Tregs and negatively correlated with cancer-suppressing immune cells, such as CD8 T cells and NK cells (Fig. 9B). We divided cervical cancer patients into two groups according to the median DDOST expression. The Treg cell infiltration level was higher, while the infiltration levels of CD8 T cells and NK cells were lower in the high DDOST expression group (Fig. 9C).

### Association of DDOST with drug sensitivity

**Fig. 10:**
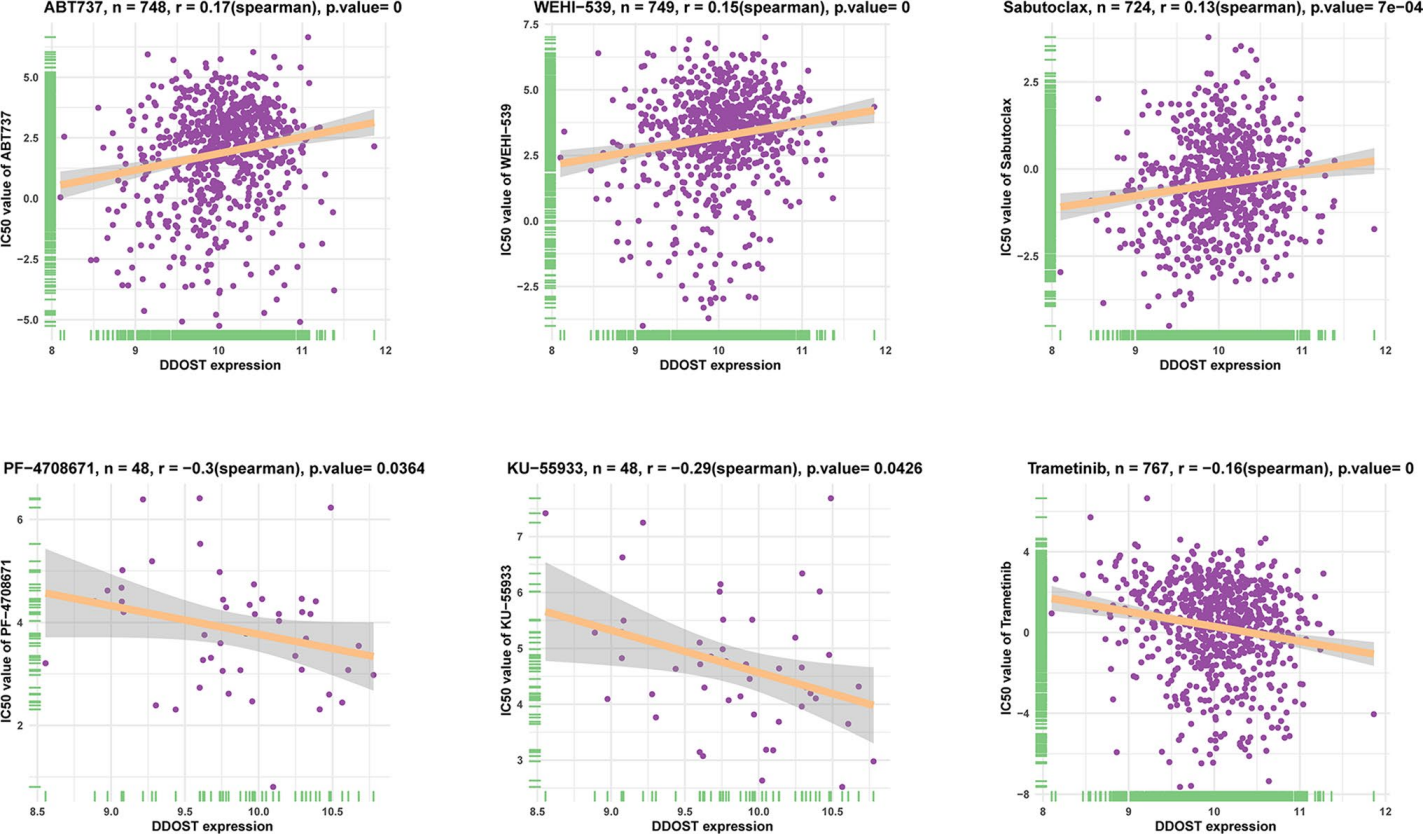
Drug resistance analysis of DDOST. The correlation between DDOST expression and IC50 values of indicated anti-cancer drugs. P = 0 means p < 0.0001

We analyzed the relationship of DDOST and drug resistance to provide a suitable medication selection for patients with high expression DDOST. The results indicated that, among 192 anti-tumor drugs in GDSC database, DDOST expression was positively correlated with the IC50 of 32 drugs, such as ABT737, WEHI-539, Sabutoclax, cisplatin, oxaliplatin, and gemcitabine, and negatively correlated with the IC50 of 11 drugs, such as PF-4708671, KU-55933, dasatinib, and Trametinib (Fig. 10, Supplementary Table 1). The above results suggested that DDOST was highly likely to be applied in the clinic as a drug target.

### Experimental verification of DDOST expression in cervical cancer

**Fig. 11:**
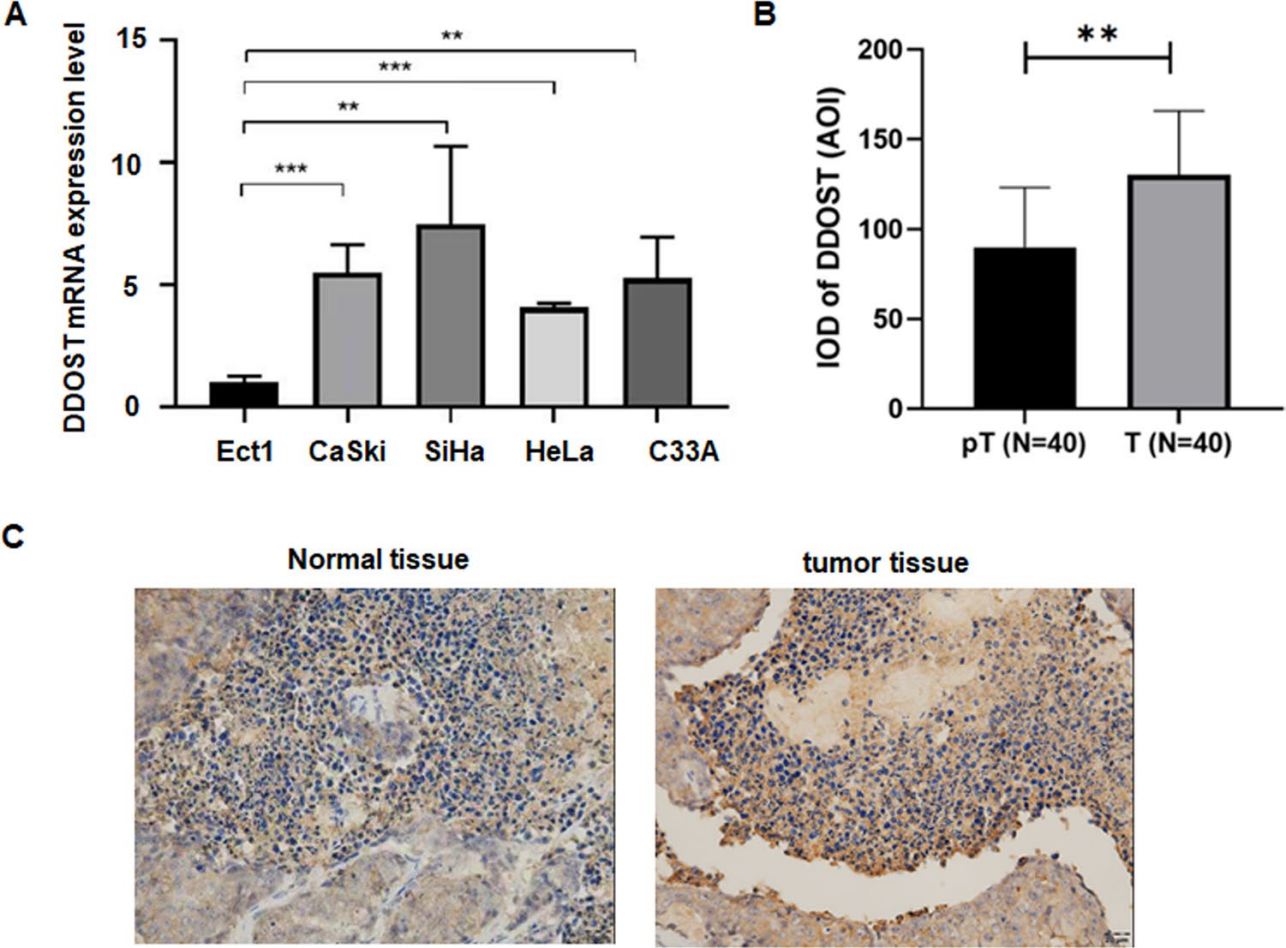
Experimental verification of DDOST expression in cervical cancer **A**, The expression of DDOST in indicated cell lines via qRT-PCR assay. **B**–**C**, The protein expression of DDOST in cervical and tumor tissues via immunohistochemical assay

Finally, we conducted experimental validation on the expression of DDOST in cervical cancer. Through qPCR detection, we found that the expression of DDOST was higher in tumor cell lines than in normal cell lines (P < 0.01, Fig. 11A). Through immunohistochemistry, we found that the protein level of DDOST was higher in tumor tissues than in adjacent non-tumor tissues in 40 cervical cancer patients (Fig. 11B, C). These results validate the findings of bioinformatics analysis.

## Discussion

Glycosylation, the attachment of carbohydrates to polypeptides, is an important form of protein post-translational modification in eukaryotes and is present in more than half of proteins [23–25]. Evidence suggests that glycosylation of proteins is non negligible in the process of tumor progression [26–28]. In particular, OST-mediated protein glycosylation is a key player in signal transduction, protein folding, and degradation [29, 30]. It has also been confirmed that glycosylation of protein is closely related to immune escape of tumor cells [31–33]. DDOST, a subunit of the OST complex, plays a critical role in N-glycosylation. Due to that there are no researches on DDOST in cervical cancer, in the current study, we explored the role of DDOST in cervical carcinogenesis.

One study used integrated bioinformatics analysis and validated that DDOST was correlated with progression and growth in colon adenocarcinoma [34]. DDOST expression was increased and correlated with tumor metastasis in cutaneous squamous cell carcinomas [35]. Endocytosis-associated protein SCAMP3 was highly expressed and associated with poorer survival and progression in hepatocellular carcinoma (HCC). DDOST expression was positively associated with SCAMP3 in HCC patients [36]. One group reported that DDOST was involved in breast oncogenesis and could be a valuable biomarkers for breast cancer development [37]. Suppression of ribophorin 1 triggered endoplasmic-reticulum-stress-induced apoptosis in part via upregulation of DDOST in breast cancer [38]. Downregulation of DDOST reduced cell proliferation in hepatocellular carcinoma (HCC) [39]. Moreover, DDOST could be a prognostic biomarker and correlated with immune infiltrates in HCC [40]. One study showed that DDOST associated with malignancies and immune TME in gliomas [41]. DDOST may serve as an unfavourable biomarker for gliomas [42]. In view of this, our study comprehensively investigated role of DDOST in pan-cancer and cervical cancer. Joint analysis of TCGA and GTEx databases revealed that DDOST was significantly upregulated in 27 of 33 tumor types, including cervical cancer. Experimental verification of DDOST expression in cervical cancer cells and tumor tissues confirmed this conclusion.

Zhu et al. found that high expression of DDOST was linked to poorer survival in HCC patients. DDOST was linked to cell cycle and immune response by the PPAR pathway. Specifically, DDOST expression was associated with immune infiltration of Th2 cell and cytotoxic cells [40]. Similarly, DDOST was revealed to be associated with worse prognosis in glioma patients. DDOST was correlated with immune microenvironment in gliomas, which was related to infiltration of B cells, CD4 + T cells, tumor-associated macrophages and CAFs [41]. MELK (maternal embryonic leucine zipper kinase) was elevated in glioma tissues and associated with poorer survival. DDOST induced the tumor immunosuppressive microenvironment in gliomas [43]. The TME is the cellular environment in which tumor cells reside, and its composition includes extracellular matrix, soluble molecules, and tumor stromal cells [44]. Using single cell expression profiling of cervical cancer, we revealed that DDOST was universally expressed in TME. Among TME components, the most highly expression of DDOST was in malignant. We also analyzed the correlation of DDOST with immune cell infiltration in the TME of cervical cancer. DDOST expression in cervical cancer is positively correlated with cancer-promoting immune cell Tregs and negatively correlated with cancer-suppressing immune cells, such as CD8 T cells and NK cells. These results indicated that patients with high expression of DDOST may be in immunosuppressive TME, which may be one of the main reasons that DDOST causes the poor prognosis of patients.

We evaluated the relationship between DDOST expression and patient prognosis in a pan-cancer. Cox regression analysis of 33 tumor types showed that the expression of DDOST was significantly associated with prognosis in multiple cancer types, especially cervical cancer. Cervical cancer patients with high expression of DDOST had worse OS, DSS, DFI, and PFI. Furthermore, Kaplan–Meier survival curves showed that in 16 tumor species, DDOST was a high-risk factor for OS, including cervical cancer. These results indicated that DDOST was a potential oncogene and risk factor in cervical cancer and most tumors. GSVA and GSEA revealed that DDOST was most strongly associated with glycolysis, unfolded protein response, PI3K/AKT/mTOR signaling, and most cell cycle-related pathways. It is known that PI3K/AKT/mTOR pathway is involved in TME and regulation of immune response [45, 46]. These results suggest that DDOST may have a complex role in influencing the cell cycle and TME. Next, we explored the correlation between DDOST expression and the TME-related signatures in pan-cancer. DDOST expression was significantly correlated with various pathway scores in pan-cancer, among which were mainly DNA damage repair and EMT related pathways. Finally, drug sensitivity analysis also suggests that DDOST could be a potential target for anti-cancer therapy.

Taken together, our findings suggest that DDOST could serve as a prognostic biomarker in cervical cancer as well as in a variety of tumors. High DDOST expression confers poor prognosis of tumor patients. In addition, the expression of DDOST is closely related to the immunosuppressive TME. It is required to explore the function of DDOST in cervical cancer cells, including cell proliferation, apoptosis, cell cycle, migration and invasion. Moreover, in vivo study is necessary to determine the role of DDOST in cervical tumorigenesis. Furthermore, the mechanism of DDOST-involved cervical oncogenesis needs to be clarified. These findings may help elucidate the role of DDOST in tumorigenesis and progression and provide a reference for achieving precision cancer therapy and personalized immunotherapy in the future.

### Supplementary Information

Supplementary file1: (CSV 13 KB)

## Data Availability

All the data will be provided on reasonable request from the corresponding author.

## Abbreviations:

NK cells: Natural killer cells
PPP1CC2: Phosphoprotein phosphatase 1 catalytic subunit gamma 2
PCR: Polymerase chain reaction
FBS: Fetal bovine serum
DMEM: Dulbecco's Modified Eagle Medium
RPMI: Roswell Park Memorial Institute
PI3K: Phosphoinositide 3-kinase
mTOR: Mammalian target of rapamycin
TME: Tumor microenvironment
EMT: Epithelial–mesenchymal transition
SCAMP3: Secretory carrier membrane protein 3
PPAR: Peroxisome proliferator-activated receptor
CAFs: Cancer-associated fibroblasts
